# Socioeconomic Status and Health-Compromising Behaviour: Is it All About Perception?

**DOI:** 10.5964/ejop.v16i3.1840

**Published:** 2020-08-31

**Authors:** Natascha de Hoog, Susanne van Dinther, Esther Bakker

**Affiliations:** a Faculty of Psychology, Open University of the Netherlands, Heerlen, the Netherlands; Connection Lab, San Francisco, CA, USA

**Keywords:** socioeconomic status, perception, subjective, health-compromising behaviour, social identity

## Abstract

Socioeconomic status (SES) is associated with many health issues and health-compromising behaviour (HCB). Most research is based on objective indicators of SES, even though subjective SES, someone’s perception of their social standing, is also related to health. Moreover, perceptions of health and HCB might also be of importance. Therefore, this study examined the relationship between both objective and subjective SES and perceived health and HCB respectively, and the role of perceptions of HCB. 326 respondents completed measures of objective and subjective SES, perceived health, HCB and perceptions of HCB. Results showed objective and subjective SES were related to perceived health. Only subjective SES was related to HCB, while for objective SES a moderating effect of perceiving HCB as typically high or low SES was found. Not only objective SES, but especially perceptions of SES and HCB are associated with someone feeling healthy and engaging in HCB. Health interventions should try to tackle perceptions of SES and HCB, either by invalidating current SES related perceptions or by emphasizing new healthy perceptions.

Social inequality in health is described as a global problem for people’s health (Siegrist & Marmot, 2004). It is perceived as a complex issue that can be summarized by the fact that the health of people, and even their life expectancy, depends in part on where in the world they are born. Social determinants of health mainly have to do with poverty (Marmot, 2005). Poverty not only refers to how much money one has, but also lack of accessibility to resources like proper nutrition, clean drinking water or good medical care (Adler et al., 2016). Available resources are unequally distributed affecting many different less-advantaged groups based on ethnicity, gender, sexual orientation or simply place of residence (Adler & Rehkopf, 2008). Over the past decades systematic disparities in health and mortality have increased between people with a high position and people with a low position in the community (Mackenbach et al., 2008). This position is partly determined by one’s social and economic place in the social hierarchy (Marmot, 2006), often referred to as socioeconomic status (SES).

SES is negatively associated with a range of health outcomes, including chronic illness, sight and hearing problems, mental disorders and addictions (Costa-Font & Hernandez-Quevedo, 2012; Dalstra et al., 2005; Marmot, 2005). People with a low SES are less likely to rate their health as good (Mackenbach et al., 2008) and, above all, have a lower life expectancy (Singh & Siahpush, 2006; van Lenthe et al., 2004). Studies showed low SES is also related to health-related behaviour like smoking, unhealthy food habits and an inactive lifestyle (Hanson & Chen, 2007; Lynch, Kaplan, & Salonen, 1997; Senn, Walsh, & Carey, 2014;). Health-related behaviour refers to any behaviour that has or is believed to have an important effect on health. One can differentiate between health promoting behaviour when talking about health-related behaviour that improve health, and health-compromising behaviour for behaviour that has a negative effect on health.

The relationship between SES and health and health-related behaviour is well established. However, less is known about the factors contributing to this relationship (Costa-Font & Hernandez-Quevedo, 2012), especially those that could be influenced easier than it is to change someone’s SES. In this paper we address ways in which subjective perceptions of SES, health and health-compromising behaviour could be part of the explanation.

## Subjective SES, Health and Health-Related Behaviour

Most research conducted so far is based on objective indicators of SES such as income, education and occupation (Operario, Adler, & Williams, 2004), although other indicators have been used as well (Galobardes, Shaw, Lawlor, Lynch, & Smith, 2006). However, subjective measurements that include one’s individual perception also play an important role in health-related research (Adler, Epel, Castellazzo, & Ickovics, 2000). Subjective SES, an individual’s perception of their relative position in the social hierarchy, has been associated with health status and health-related behaviour independent of objective socioeconomic indicators (Ghaed & Gallo 2007; Reitzel et al., 2011): Low subjective SES is associated with lower perceived health and more health-related behaviour. For instance, subjective SES is related to behaviour like smoking, physical activity and fruit and vegetable consumption (Ghaed & Gallo, 2007; Reitzel et al., 2011), and, consequently, is related to a person’s health.

Subjective SES has been shown to correlate with objective measures of SES, such as income, education and occupation, but it can also be used as an independent measure of an individual’s SES (Ostrove, Adler, Kuppermann, & Washington, 2000). Singh-Manoux, Marmot, and Adler (2005) tested the predictive ability of both types of SES on changes in health status. Analyses revealed that change in health over a 3-year period is socially patterned, which means that lower SES was associated with poorer health, whereas people with a higher SES showed either an improvement or no deterioration. However, this relationship was only significant for the subjective measure of SES, and this emphasizes the additional value of individual perception and subjective measurements in this type of research. Singh-Manoux et al. (2005) even suggest subjective SES might be a stronger predictor than objective SES, because subjective ratings more accurately capture subtle aspects of social status (Adler et al., 2000).

Another reason that can show how subjective SES might be important in explaining differences in health and health-related behaviour lies in the fact that subjective SES taps into the extent to which one sees oneself as someone with a low or high SES. Identifying with certain groups or categories can influence how one feels and behaves, as proposed by social identity theory (Tajfel & Turner, 1986). The theory states that an important component of the self-concept is derived from belonging to one or more social groups and categories (Abrams & Hogg, 1990). People who identify strongly with a group will tend to adopt the norms, attitudes and behaviour sanctioned by group members (Terry & Hogg, 1996). Thus, it is more about an individual’s own subjective perspective belonging to a group rather than an objective measure. Further, the theory states that when an identity becomes more important for one’s self-concept, one is more likely and more motivated to behave in a way that is congruent to this identity (Stets & Burke, 2003; Stryker & Burke, 2000). By acting in identity-congruent ways and, moreover, by social interaction, attitudes and beliefs will be confirmed through which they become an even more important part of one’s identity (Stets & Burke, 2003).

From this perspective, SES can be seen as something that can become an important part of one’s identity. Someone can identify with the group of persons with a low or high SES, as indicated by their subjective SES. Consequently, SES will become relevant to how they see themselves, and different aspects of their life like their health, as well as how they behave. Thus, subjective SES is proposed to be related to both perceived health and health-related behaviour. In order to examine this proposed relationship between subjective SES and health it is important to not look at objective, but at subjective health, also known as perceived health.

## Perceptions of Health and Health-Related Behaviour

Objective health is often determined by objective factors such as blood pressure, weight and mortality rates. However, subjective health, which is usually termed perceived health, is a subjective relative measure of overall health status and may include aspects that are difficult to capture clinically, such as incipient disease, disease severity, physiological and psychological reserves. When people rate their health, they think not only of their current situation, but also of trajectories, declines and improvements (Statistics Canada, 2010). Therefore, perceived health is thought of as a more comprehensive measurement approach (Sadiraj & Groot, 2006). Despite the subjective nature of this measure, indicators of perceived health have been found to be a good predictor of people’s future health care use and mortality (Idler & Benyamini, 1997; Miilunpalo, Vuori, Oja, Pasanen, & Urponen, 1997). Perceived health has been shown to predict mortality, even after adjustment for key covariates as functional status and co-morbidity (DeSalvo, Bloser, Reynolds, He, & Muntner, 2006).

In the case of health-related behaviour, perception might play an even more important role. Not only do perceptions differ as to what qualifies as health-related behaviour (Conner, 2001), but also whether those behaviours are typical of certain groups, like low and high SES (Oyserman, Fryberg, & Yoder, 2007). A study by Bowen, Hickman, and Powers (1997) illustrates the impact of perception of oneself and of typical group behaviour on actual behaviour. They found that African American women who identified as ‘African American’ were more likely to seek mammography screening than those who identified as ‘Black’. Bowen and colleagues suggest that women who identified as African American felt less at odds with the dominant culture than those who identified as Black. In addition, several studies by Oyserman et al. (2007) showed that racial-ethnic minority and, more relevant for our current study, low SES Americans, viewed health promoting behaviour as more characteristic of White and middle-class society than as characteristic of their in-group. They showed a moderating effect of classification of behaviour, with participants who considered themselves low SES less likely to engage in health promoting behaviour, and they were more likely to view health promoting behaviour as typical for people with a high SES.

Thus, there is some evidence that certain health-related behaviour can be seen as typical behaviour for a specific group (Oyserman et al., 2007), such as the group of low or high SES. Reasoned from social identity theory (Tajfel & Turner, 1986), this classification can have consequences for actual behaviour. An individual is more likely to demonstrate a health-related behaviour that is appropriate to the behavioural norms of the group, which they identify with. Simultaneously, one is more likely to avoid performing a health-related behaviour that is appropriate to the behavioural norms of a group they do not identify with (White, Simpson, & Argo, 2014). In other words, individuals who consider themselves to be low SES could perceive health promoting behaviour as not fitting with their own identity and this incongruence could impede them from performing this behaviour. Therefore, they are more likely to smoke, drink too much alcohol, be physical inactive or eat unhealthy food. In addition, they are less likely to feel health promotion efforts are relevant to them (Oyserman et al., 2007). Thus, health promotion is a social, rather than an individual dictated choice. One is more likely to behave in congruence with the preferences and (implicit) prescribed lifestyle of the group they identify with. Therefore, it was expected that the relationship between SES and health-related behaviour would be stronger when perceptions of the health behaviour are congruent with one’s SES.

## Current Study

Although research that examines both objective and subjective measures of SES as predictors of health is growing recently (Hoebel, Müters, Kuntz, Lange, & Lampert, 2015), the exact relationship between these variables and health and health-compromising behaviour remains unclear. In addition, little is known about people’s perceptions of health-related behaviour and how these perceptions relate to SES, health and actual behaviour. Most studies on the topic have investigated only specific populations (e.g., Miyakawa, Hanson, Theorell, & Westerlund, 2012). More importantly, up until now no research has examined these relationships from a subjective perspective, combining measures of objective and subjective SES, as well as subjective measures of health and perceptions of health-compromising behaviour in a single study. The goal of this research is to better understand the relationship that exists between SES, health, health-related behaviour and perceptions thereof. Understanding these relationships could be valuable in determining which type of SES measures are most related to an individual’s perceived health and health-related behaviour. This could potentially offer insight into how to intervene in or prevent negative health outcomes as a product of these variables. Therefore, this study examined the relationship between both objective and subjective SES and perceived health and health-compromising behaviour respectively, and the role of perceiving health-compromising behaviour as typically high or low SES.

The following hypotheses were postulated:

1. Both objective and subjective SES is positively correlated with perceived health. The higher a person’s SES the better they will perceive their health.

2. Both objective and subjective SES is negatively correlated with health-compromising behaviour. The lower a person’s SES the more health-compromising behaviour they will perform.

3. Perceptions of health-compromising behaviour moderates the relationship between both objective and subjective SES and health-compromising behaviour. The relationship should be stronger when perceptions of the health-compromising behaviour corresponds with someone’s SES.

## Method

### Participants and Procedure

Respondents were Dutch men and women over 18 years old from the general population. The minimum number of respondents needed to test our hypotheses was 110 (104 + 6 variables; Tabachnick & Fidell, 2001). Approximately 500 people from the researchers’ social and professional networks were approached by mail and on several Facebook groups with the invitation to participate in the study. Due to time and financial restraints it was not possible to randomly select a representative sample of the general population, but every effort was made to make sure respondents with both low and high socioeconomic status participated in the study by approaching as many different groups as possible. For example, employees from a company with mainly low-level employees were approached, as well as undergraduate students from an online university. The invitation contained a brief introduction about the purpose and content of the study, that is, an online survey about social status and the relationship with health and health-related behaviour, and provided a link to the survey. No compensation for participation was offered. At the start of the survey, participants were informed that participation was voluntarily and anonymous. After providing consent, the survey was automatically started. A total of 369 respondents participated in the survey, of which 43 respondents were excluded because they preferred to conceal their income, whereby determination of their objective SES was not possible. The remaining 326 respondents consisted of 98 men and 228 women in the age from 19 to 80 (*M* = 44.44, *SD* = 15.18).

### Measures

A cross-sectional questionnaire was chosen for time and financial reasons, and because we first wanted to test our predictions before attempting more elaborate designs like a longitudinal study. The questionnaire included (in order) measures of age, gender, objective SES, subjective SES, perceived health, health-compromising behaviour and perceptions of health-compromising behaviour. In order to get the most valid and best comparable results, measures were chosen that were used in previous research on the same topic whenever possible. When no useful existing measures were found, new measures were created building on the literature as much as possible.

#### Objective SES

Objective SES was operationalized as a combination of education, occupation and income level, where a higher combined level reflects a higher SES. It was measured with three items adapted and translated from Adler et al. (2000). Measures of education, income and occupation served as indicators of objective SES. Participants were asked to report their highest completed education level, with answer categories ranging from (1) *no completed education* to (7) *university degree*. Occupation was assessed with categories ranging from (1) *no paid occupation* to (6) *academic or professional occupation.* Participants were asked about their annual income with answer categories ranging from (1) *€0 - €10.000* to (6) *> €100.000.* A composite measure of education, occupation and income was created by standardizing each variable and taking their mean (Adler et al., 2000). Cronbach’s alpha was .59.

#### Subjective SES

Subjective SES was operationalized as one’s own perceived social status compared to others, which was assessed with a translation of the MacArthur scale of Subjective Social Status (Ostrove et al., 2000). Participants were asked to imagine a ladder with 10 rungs and to indicate, with a number between 1 and 10, where they thought they fell on the ladder ranging from (1) *people who are the worst off in their community* to (10) *people who are the best off in their community*.

#### Perceived Health

Perceived health was operationalized as how good one perceives their own personal health. Participants were asked to rate their health with a number between 1 (*poor*) and 100 (*excellent*). A substantial body of international research has reported the item to be significantly and independently associated with specific health problems, use of health services, mortality and sociodemographic characteristics, and is judged to be appropriate for use in population surveys (Bowling, 2005).

#### Health-Compromising Behaviour (HCB)

HCB was operationalized as a combination of a number of health-related behaviours that have been shown to be beneficial or detrimental to health, with a higher combined score indicating more engagement in HCB. An index of health-compromising behaviour (HCB) was created based on prior research on multiple HCB, which included substance use, physical activity, diet, sleep and relaxation. All of which have been shown to be related to SES (Hanson & Chen, 2007; Lynch et al., 1997). An example-item is; ‘*I exercise, walk or cycle at least 30 minutes a day*’. Participants answered on a 5-point Likert scale ranging from 1 (*always*) to 5 (*never*). Answers to the negative HCB (e.g., smoking) were recoded and a mean score of the 10 items was calculated. A higher score meant more HCB. Cronbach's alpha was .60.

#### Perceptions HCB

Perceptions of HCB were operationalized as the extent to which one perceives HCB as something typically for low or high SES. Participants were asked to rate the 10 HCB on a 5-point Likert scale ranging from 1 (*typical behaviour for low SES*) to 5 (*typical behaviour for high SES*). These HCB correspond to the questions from the HCB scale. The negative HCB items were recoded and the mean score was calculated. A higher score meant that respondents perceived the HCB as typical behaviour for persons with a low SES. Cronbach’s alpha was .77.

### Analyses

Analyses were conducted using SPSS (Version 22). After recoding negative formulated items, reliability analyses were performed and mean scores, standard deviations and skewness of all scales were calculated. In accordance with West, Finch, and Curran (1995), variables all had a skewness < 2, which can be considered as normally distributed. Then correlations between all variables were calculated. All variables were standardized before testing the hypotheses in order to better compare the different scales. Hypothesis 1 and 2 were tested using multiple regression analysis. To test Hypothesis 3, hierarchical multiple regression analysis (HMRA) was conducted, including a dummy variable for gender (0 = male, 1 = female) and two interaction-terms (multiplications of the independent variables with the moderator). All analyses controlled for gender and age to prevent confounding. The correlation between objective SES and subjective SES was .50, but there was no indication of multicollinearity, therefore, both measures of SES were simultaneously included in all analyses. Scatterplots and Q-Q plots were used to test the other assumptions of regression analysis, which were all satisfactory.

## Results

### Descriptive Statistics and Correlations

Before testing the hypotheses, descriptive statistics of the study population and correlations between all main variables were calculated (see Table 1). Almost two-thirds of the study population consisted of persons who had a college (37.7%) or university degree (23.9%). The distribution of occupational levels and income levels on the other hand, were more evenly spread across all categories, which made for a more even distribution of objective SES scores. On subjective SES, respondents rated themselves on average as slightly above the midpoint of the SES scale (*M* = 6.22, *SD* = 1.60; range 1-10). However, 28.2% of the participants rated themselves with a score of 1 through 5 and could be considered ‘low’ subjective SES, while the other 71.8% rated themselves with a score of 6 through 10 and could be seen as ‘high’ subjective SES. Respondents perceived HCB relatively often as typically low SES. When looking at individual HCB, at least 30 minutes of daily exercise, 30 minutes of relaxation and daily eating of fruit, vegetables and fresh prepared meals, were perceived as typically high SES. In contrast, smoking, substance and alcohol use were perceived as typically low SES. Daily sitting longer than eight hours and getting enough sleep were not perceived as typically high or low SES. Respondents reported moderate scores on engaging in HCB (*M* = 2.15). Mean scores on perceived health, measured on a scale from 1 to 100, fell above the midpoint of the scale (*M* = 77.13), but the high standard deviation (*SD* = 14.50) indicates that there was a lot of variation in scores.

**Table 1:** Descriptive Statistics and Correlations of all Variables (N = 326)

Variable	Range	*M*	*SD*	1	2	3	4	5	6
1. Gender	-	-	-	-					
2. Age	19-80	44.44	15.18	-.25***	-				
3. Objective SES	2-2	0.00	0.74	-.12*	-.15**	-			
4. Subjective SES	1-10	6.22	1.60	-.12*	.04	.50***	-		
5. Perceived health	1-100	77.13	14.50	.12*	-.08	.22***	.24***	-	
6. HCB	1-5	2.15	0.48	-.21***	-.23***	-.11	-.19***	-.31***	-
7. Perceptions of HCB	1-5	3.44	0.46	-.08	.01	.00	.01	.02	.09

*p < .05. **p < .01. ***p < .001.

When checking for potential confounding, both age and gender were found to be related to SES and HCB. Age correlated significantly to both objective SES, *r* = -.11, *p* = .02, and HCB, *r* = -.23, *p* < .001. Therefore, age was included in all analyses as a confounder. Men rated their subjective SES as higher (*M* = 6.52, *SD* = 1.80) than women *(M* = 6.10, *SD* = 1.48), *t*(156.84) = 2.06, *p* = .04. Men also had a higher (*M* = 0.13, *SD* = 0.79) objective SES than women (*M* = -0.06, *SD =* 0.72), *t*(324) = 2.08, *p* = .04. However, women rated their perceived health as higher (*M =* 78.23, *SD* = 13.64) than men (*M* = 74.58, *SD* = 16.11), *t*(324) = -2.09, *p* = .04. Finally, men reported higher scores on performing HCB than women (respectively; *M* = 2.30, *SD* = 0.59; *M* = 2.08, *SD* = 0.40), *t*(136.40) = 3.34, *p* < .01. Therefore, gender was also controlled for in all analyses.

As can be seen in Table 1, subjective SES was significantly related to the composite measure of objective SES, *r* = .50, *p* < .001, suggesting that participants take into account their relative standing on the various components of SES when rating their standing on the ladder. Significant correlations were also found between both forms of SES and perceived health, *r* = .24, *p* < .001 and *r* = .22, *p* < .001, respectively. Only subjective SES showed a significant negative correlation with HCB, *r* = -.19, *p* < .001. For objective SES this correlation was negative, but non-significant, *r* = -.11, *p* = .053. Finally, both forms of SES were not significantly correlated to perceptions of HCB as typically low SES, *r* = .00, *p* = .998 and *r* = .01, *p* = .92, respectively.

### Hypotheses Testing

Hypothesis 1 assumed a positive relationship between both forms of SES and perceived health. The multiple regression-analysis showed objective SES and, to a slightly higher extent, subjective SES, were significantly related to perceived health, *B* = 0.13, *t*(321) = 2.11, *p* = .04 and *B* = 0.20, *t*(321) = 3.15, *p* = .002, respectively. Together with gender and age, objective and subjective SES explained a significant part of the variance in perceived health, *R*^2^ = .10, *F*(4, 325) = 8.52, *p* < .001.

The second hypothesis, that assumed a negative relationship between SES and HCB, was tested using the same multiple regression-analysis only with HCB as dependent variable (see Table 2). Results indicated that subjective SES, contrary to objective SES, was related to the extent of performing HCB, *B* = -0.16, *t*(321) = -2.64, *p <* .01 and *B* = -0.12, *t*(321) = -1.91, *p =* .06, respectively. 18.5% of the variance in HCB is accounted for by gender, age, objective and subjective SES, *F*(4, 325) = 18.20, *p* < .001.

**Table 2:** Results of the Hierarchical Multiple Regression-Analysis on Health-Compromising Behaviour (N = 326)

Independent variable	Health-compromising behaviour		
	*B*	*SE*	β
Step 1 (*R*^2^ = .13***)			
Constant	0.44***	0.10***	
Gender	-0.63***	0.12***	0.29***
Age	-0.30***	0.05***	0.30***
Step 2 (*R*^2^ = .19***, Δ*R*^2^ = .06***)			
Constant	0.49***	0.10***	
Gender	-0.70***	0.12***	0.32***
Age	-0.32***	0.05***	0.32***
Objective SES	-0.11	0.06	0.11
Subjective SES	-0.16**	0.06**	0.16**
Perceptions	0.07	0.05	0.07
Step 3 (*R*^2^ = .29***, Δ*R*^2^ = .10***)			
Constant	-0.48***	0.09***	
Gender	0.68***	0.11***	0.31***
Age	0.32***	0.05***	0.32***
Objective SES	0.07	0.06	0.07
Subjective SES	0.15**	0.06**	0.15**
Perceptions	-0.06	0.05	-0.06
Objective SES x Perceptions	-0.30***	0.06***	-0.33***
Subjective SES x Perceptions	-0.02	0.06	-0.03

*Note.* All variables are standardized variables. Gender: male = 0, female = 1.

**p < .01. ***p < .001.

Finally, Hypothesis 3 predicted that perceptions of HCB moderates the relationship between both objective and subjective SES and HCB. Results of the HMRA are represented in Table 2. Gender and age were entered in the first step of the regression-analysis and explained 13.1% of the variance in performing HCB. In step 2, objective and subjective SES and classification of HCB were entered, and they explained a significant additional variance in performing HCB, Δ*R*^2^ = .04, Δ*F*(2, 321) = 24.36, *p* < .001. Subjective SES, contrary to objective SES, was related to HCB, *B* = -0.16, *t*(320) = -2.64, *p <* .01 and *B* = -0.11, *t*(320) = -1.90, *p =* .06, respectively. Perceptions of HCB had no direct relationship with HCB, *B* = .07, *t*(320) = 1.35, *p =* .18. In the final step, the interaction terms Objective SES x Perceptions and Subjective SES x Perceptions were entered, and this explained a significant increase in variance in HCB, Δ*R*^2^ = .10, *F*(2, 318) = 23.01, *p* < .001. Only the interaction of Objective SES x Perceptions was significant *B* = -0.30, *t*(318) = -5.31, *p <* .001. Thus, perceptions of HCB was a significant moderator of the relationship between objective SES and HCB. The standardized simple slope for persons 1 *SD* below the mean of perceptions of HCB as typically behaviour for low SES was β = -0.21, *p* < .01 and the standardized simple slope for persons 1 *SD* above the mean was β = 0.37, *p* < .001. This means that there is a negative relationship between objective SES and HCB when HCB is perceived as typical behaviour for persons with a low SES, and there is a positive relationship between objective SES and HCB when HCB is seen as typical for high SES.

## Discussion

This research examined the relationship between both objective and subjective SES and perceived health and HCB respectively, and the role of perceiving HCB as typically high or low SES. Previous research revealed SES to be related to health as well as HCB (Dalstra et al., 2005; Hanson & Chen, 2007), and showed that perceptions of behaviour correspond with individuals’ actual behaviour and consequently their health (Oyserman et al., 2007). However, so far no research was conducted examining these different constructs all in one study. This study showed that objective SES and subjective SES both were related to perceived health. Only subjective SES was related to HCB. Further, partial support was found for the predicted importance of individual perception in the form of perceptions of HCB. The moderating effect of perceiving HCB as typically high or low SES was only present in the relationship between objective SES and HCB.

The current study showed a correlation of .50 between objective and subjective SES, which is consistent with prior research that found correlations ranging from .20 to .50 (Ostrove et al., 2000). This suggests that, besides objective indicators, there are more factors related to someone’s subjective SES. At the same time, it confirms that although both forms of SES are related, it concerns two different constructs and it emphasizes the importance of including subjective measures in research. Subjective measures compass more aspects that are not measurable explicitly. Adler and Stewart (2007) found that people take into account factors like personal values, ethics, and altruistic actions, when they determine their subjective SES.

The first hypothesis, that presumed a relationship between SES and perceived health, was supported. The results show that the lower someone’s SES, the lower their perceived health, and vice versa. This finding is in line with earlier studies (Mackenbach et al., 2008; Ostrove et al., 2000). Both forms of SES were equally strong related to perceived health, which indicates that subjective SES could be a relevant factor in research on SES and perceived health. We only measured perceived health, but we would expect a similar relationship between SES and objective health measures, as found in previous studies (Dalstra et al., 2005; Singh-Manoux et al, 2005). Interestingly enough, Singh-Manoux et al. (2005) only found subjective SES to be predictive of objective health over a three-year period, suggesting perceptions to have an important influence on objective health outcomes.

Further, Hypothesis 2 that assumed a negative relationship between SES and HCB was partly supported. Only subjective SES had a significant relationship with HCB, which means that the lower a person’s subjective SES, the more they will engage in health-compromising behaviour, and vice versa. This indicates that there are other subjective factors beyond objective measures like income, education and occupation that are associated with HCB (Ghaed & Gallo, 2007). It seems like it is not so much being low or high SES that is related to one’s health-related behaviour, but whether or not one sees oneself as belonging to a certain SES group (Abrams & Hogg, 1990). Therefore, subjective SES should always be included in developing effective health interventions.

Finally, it was assumed that the relationship between SES and HCB would be stronger if HCB was perceived as typically of someone’s SES. This study found partial evidence for this prediction. Whereas only subjective SES had a direct relationship with HCB, moderation by perception of HCB was only found for objective SES. Objective SES was negatively related to HCB when HCB was perceived as typical behaviour for persons with a low SES, and positively related when it was perceived as typical behaviour for persons with a high SES. Thus, if one sees HCB as typical for low SES (Terry & Hogg, 1996), one is more inclined to perform HCB when one is low SES and less inclined when one is high SES, and the reverse is true when one sees HCB as typical for high SES.

The prediction that perceptions of HCB are related to one’s behaviour, is herewith supported. Nevertheless, it is interesting that this moderating effect was only found for objective and not for subjective SES, because subjective SES is determined by one’s own individual perspective of their position in the social hierarchy. Thus, one explicitly perceives oneself as belonging to the group low or high SES. For this reason, it was expected that perceptions of HCB would be related to HCB. One’s SES is related to behaviour, because it contains aspects of the role, status and expectations that are connected to membership of a specific social group (Weyers, Dragano, Richter, & Bosma, 2010). An individual is more likely to perform a health-related behaviour that is appropriate to the behavioural norms of the group, which they identify with (White et al., 2014). Possibly the identification with the group of persons with a low or high SES was not salient enough in this study through which no moderating effect was found for subjective SES. People may belong to many different types of social groups and consequently can have multiple different social identities. Except ‘low or high SES’ one can also identify with, for example, ‘women’, ‘Christians’ or ‘Americans’. The social identity that is salient at a certain moment determines the norm and thereby the actual performing of behaviour (Haslam, Jetten, Postmes, & Haslam, 2009). It is imaginable that identification with another social group than either low or high SES is more important in the association with performing HCB for persons who perceive this as behaviour for the group with high SES. Alternatively, it could be that perceptions of one’s SES already incorporate notions about typical norms and behaviours associated with SES, including engaging in HCB. Therefore, perceptions of HCB could not have any additional influence in this case.

Interestingly enough, it seems it is mainly perceptions that are related to HCB. Only subjective SES, thus the perception of belonging to a particular SES group, was directly related to HCB. Objective SES by itself was unrelated to HCB, unless those persons perceived HCB as typical for someone’s SES. This once again emphasizes the importance of focusing on people’s perceptions and subjective factors in trying to understand why such differences in health and health-related behaviour exist between people of different social standing. It is important to note, however, that even though with subjective measures one is much better able to tap into people’s opinions of how they see aspects of (their) life, at the same time one is never quite sure how they come to their perceptions. It is entirely possible that some people rated their subjective SES much higher than their objective SES, not because they thought their status was that high, but because they did not want to see themselves as low SES. For our reasoning that perceptions of SES are related to health-related behaviour it does not matter how factual these perceptions are, although a .50 correlation between objective and subjective SES seems to suggest people’s subjective SES is based at least in part on objective criteria. Nevertheless, this study clearly indicates that health and health-related behaviour is about much more than socioeconomic background.

These study findings contribute to current psychological knowledge by showing the importance of both identity factors and subjective perceptions in health-compromising behaviour. Over the last decade more research has focused on the role of identity in health-related behaviour (e.g., Haslam et al., 2009). However, these studies have mainly looked at the role of belonging to certain groups and not so much at the impact of identity perceptions on actual health-related behaviour or vice versa (e.g., Oyserman et al., 2007). Our results clearly show that performing health-related behaviour is related to whether one sees this behaviour as typical of one’s group or not. This could be an additional factor in explaining why health behaviour interventions do not appeal to everyone (Kelly & Barker, 2016).

### Limitations and Recommendations

Although this study had several strengths, some limitations need to be acknowledged. The most important limitation is that this was a cross-sectional study, therefore, no strong claims about causality can be made. Although it is possible that poor health may lead to lower SES, there are numerous longitudinal studies suggesting the SES-health association is driven primarily by low SES leading to poorer health (Mulatu & Schooler, 2002; Preston & Elo, 1995), although some studies have also found opposite effects as well (Alvarez-Galvez, 2016). In addition, even though the focus of our study was on assessing people’s subjective perceptions of their SES, health and HCB, when relying on subjective measures one runs the risk of other psychological factors influencing responses (Senn et al., 2014). Moreover, health-related behaviours were self-reported and objective measures confirming the accuracy of these self-reported behaviours, such as accelerometer data for physical activity and sitting, were not collected. In addition, the order in which the questions were asked may have affected the results. For example, perceptions of HCB could have been affected by just having been asked about one’s SES and HCB. Further, the variables are measured with a questionnaire that partly consisted of existing validated scales, and partly consisted of self-constructed scales. Although the questionnaire was constructed and composed based on the most recent literature, construct validity cannot be guaranteed. Finally, because of technical and financial limitations, a self-selected sample was used, which possibly could have resulted in sample bias that may have influenced the results.

Although people from our sample were from different walks of life, the majority identified as high SES. In addition, even though the study did have a broad age range comparable to that of the general Dutch population, more women than men took part. This might limit the generalizability of our results. Taking these limitations into account, we do think this pattern of results with a Dutch sample can be generalized to other Western nations, although we acknowledge there can be large differences between countries what it means to be low or high SES (Alvarez-Galvez, 2016). We would expect that in societies with larger differences between what it means to be low or high SES the relationship with health and health-related behaviour to be stronger. This could especially be the case in poorer countries, where one needs a certain social standing just to be able to get basic health care. However, in that case it is also imaginable that subjective measures have less of an influence, because it is less important how you perceive your SES as it is to have the money or the status to get proper health care.

Results found in this study offer implications for further research. Future research should investigate the underlying reasons for subjective SES perceptions. Identifying contributing factors to people’s perceptions could be the first step in addressing and changing these perceptions, and indirectly, influence health-related behaviour. In addition, other related variables should be examined, such as interpersonal factors that could possibly influence the relationship between SES and health-related behaviour. For example, studies have found differences for marital status and ethnicity (Gardner & Oswald, 2004; Verhoeff, Poort, & Spijker, 2002), which might be key covariates future research should adjust for. The relationship between SES and health-related behaviour is a complex one that should be analysed in ways that honours those complexities (Alvarez-Galvez, 2016). Moreover, longitudinal research is recommended in order to investigate how relationships between the different variables develop during the course of life, so that interventions can be tailored to the individual and ultimately the social-economic disparities in health may decline.

### Conclusions

In conclusion, this study provides further evidence for the importance of both objective and subjective SES in understanding perceived health and HCB. Only subjective SES was related to HCB directly, while objective SES was related to HCB when HCB was perceived as typically high or low SES. Not only SES, but especially perceptions of SES and HCB are associated with the extent to which someone feels healthy and engages in HCB. Having a low SES, whether objective or subjective, appears to be disadvantageous for one’s health. Reducing differences in income and education between persons with a low and high SES would be a great intention, but changing someone’s objective SES in the short term is quite unrealistic. Instead, to advance health, health interventions should try to tackle perceptions of SES and HCB, either by invalidating current SES related perceptions or by emphasizing new healthy perceptions.
